# Sequence Polymorphism, Segmental Recombination and Toggling Amino Acid Residues within the DBL3X Domain of the VAR2CSA Placental Malaria Antigen

**DOI:** 10.1371/journal.pone.0031565

**Published:** 2012-02-09

**Authors:** Eldin Talundzic, Sheel Shah, Ope Fawole, Simon Owino, Julie M. Moore, David S. Peterson

**Affiliations:** 1 Department of Infectious Diseases, The University of Georgia, Athens, Georgia, United States of America; 2 Center for Tropical and Emerging Diseases, The University of Georgia, Athens, Georgia, United States of America; Université Pierre et Marie Curie, France

## Abstract

*Plasmodium falciparum* malaria remains one of the world's foremost health problems, primarily in highly endemic regions such as Sub-Saharan Africa, where it is responsible for substantial morbidity, mortality and economic losses. Malaria is a significant cause of severe disease and death in pregnant women and newborns, with pathogenesis being associated with expression of a unique variant of the multidomain *Plasmodium falciparum* Erythrocyte Membrane Protein 1 (PfEMP1) called VAR2CSA. Here, we characterize the polymorphism of the DBL3X domain of VAR2CSA and identify regions under selective pressure among placental parasites from women living in endemic western Kenya. In addition to significant levels of polymorphism, our analysis reveals evidence for diversification through intra-segmental recombination and novel mutations that likely contributed to the high number of unique VAR2CSA sequence types identified in this study. Interestingly, we also identified a number of critical residues that may be implicated in immune evasion through switching (or toggling) to alternative amino acids, including an arginine residue within the predicted binding pocket in subdomain III, which was previously implicated in binding to placental CSA. Overall, these findings are important for understanding parasite diversity in pregnant women and will be useful for identifying epitopes and variants of DBL3X to be included in a vaccine against placental malaria.

## Introduction

Malaria in pregnancy is a disease syndrome with devastating social and medical complications requiring multidimensional solutions. Despite intensified international efforts to reduce the malaria burden in the developing world, it is estimated that more than 54 million cases of malaria occur every year in reproductive age women [1]. Placental malaria (PM) results from massive sequestration of *Plasmodium falciparum* infected erythrocytes in the intervillous space of the placenta, causing severe clinical symptoms that result in maternal morbidity, low birth weight and/or neonatal death [2].

Malaria-induced morbidity and mortality is largely attributable to the cytoadherent nature of *Plasmodium falciparum* infected erythrocytes. This cytoadherence is mediated by *P. falciparum* erythrocyte membrane protein 1 (PfEMP1), a parasite protein encoded by the highly polymorphic *var* gene family and expressed at the surface of the infected erythrocytes. During pregnancy, expression of a single member of the *var* gene family, *var2csa,* mediates binding to a form of chondroitin sulfate A (CSA) unique to the placenta [3], [4], [5], [6], [7]. Several studies have shown that, after multiple pregnancies, women in malaria endemic regions develop antibodies that inhibit infected erythrocyte binding to CSA [8], [9], [10], supporting the notion that a vaccine based on placenta-sequestering parasites could provide protection against PM. Furthermore, evidence for the presence of a conserved antigen or shared epitopes in CSA-adherent parasites has been demonstrated by the fact that plasma and parasites from pregnant women from different malaria endemic regions show cross-reactivity [11]. This implies that antibody recognition is not dependent on geographical origin of the parasites and that a vaccine to protect against PM is feasible.

Interestingly, while highly polymorphic compared to most malarial antigens, *var2csa* is the most conserved PfEMP1 identified to date [3], [12], making it an attractive candidate for vaccine development. It is well-known that VAR2CSA is specifically up-regulated during PM or when selected *in vitro* for CSA binding [13], and disruption of the *var2csa* gene leads to loss or significant reduction of infected erythrocyte adhesion to CSA [14]. VAR2CSA consists of six Duffy-binding-like (DBL) domains, a large interdomain region (ID2) and a C-terminal region predicted to be cytoplasmic [15]. As reported previously, four of the six DBL domains (DBL2x, DBL3x, DBL5ε, and DBL6ε) have been shown to bind CSA [16], [17], although this is controversial [18], [19], [20]. In order to design molecular interventions against PM, it is important to determine the minimal CSA binding regions and understand how variability is distributed in the individual domains of VAR2CSA.

The DBL3X and DBL4 ε domains have been shown to be most conserved of the six DBL domains of VAR2CSA [21]. Interestingly, antibodies raised against recombinant proteins of the DBL3X domain show the most cross-reactivity with heterologous parasites compared to the other binding domains [22]. Furthermore, as suggested by Barfod et al. [23], antibody-mediated immunity is predominantly acquired by malaria-exposed women to DBL3X and DBL5. Srivastava et al. [19] demonstrated recently that a recombinant protein containing DBL1X, DBL2x and DBL3X are required to achieve similar CSA binding characteristics as the full-length VAR2CSA, and inhibitory antibodies raised against full-length VAR2CSA target principally the DBL3X domain. Lastly, Dahlback et al. [20] recently showed that expression constructs with the highest affinity binding similar to that of full-length VAR2CSA included the DBL3X domain. Collectively, these studies provide good evidence that the DBL3X domain can induce strong protective immunity, plays an important role in conferring higher binding specificity in the presence of the N-terminal domains, and owing to its sequence conservation, make it an excellent target for vaccine development.

The focus of this study was to characterize the complexity of *var2csa* genotypes within naturally infected placentae and investigate patterns of sequence polymorphism within the DBL3X domain. Here we determined the sequence of *var2csa* DBL3X amplified from Kenyan placental parasites and applied computational and molecular modeling methods to investigate polymorphism, recombination and selection within this domain.

## Results and Discussion

### Var2csa DBL3X domain shows a high degree of complexity within individuals

To enable complete characterization of *var2csa* polymorphism in placental parasite populations, the DBL3X region from 12 maternal placental blood samples was amplified. Five samples were from primigravid, two from secundigravid, and five from multigravid women. Complete sequences from an average of nine clones per sample were obtained. As a control, three independent plasmids containing the FCR3 DBL3X domain were also sequenced. These yielded identical sequences, with 100% sequence identity with the FCR3 *var2csa* DBL3X sequence in GenBank (GenBank accession number AY372123). Overall, 108 sequences were obtained, of which 79 were unique at the nucleotide level when compared to these samples and existing DBL3X sequences in GenBank (**Table 1**). Seventy-six of these 79 sequences were also unique at the amino acid level, demonstrating a very high ratio of nonsynonymous to synonymous mutations. In total there were 107 novel mutations at the nucleotide level. Most of the sequences obtained differed by 5 or more nucleotides in pairwise comparisons, indicating true unique sequences. Indeed of the 3081 possible pairwise comparisons between these sequences, only 73 demonstrated 4 or fewer nucleotide differences. The most interesting finding obtained from these data is the surprising degree of complexity observed within individual patient samples. Among the 12 placental samples, half showed nine or more unique *var2*csa DBL3X sequence types. It is clear from this work that the majority of the placental infections are highly complex with regard to *var2csa* sequence types, arguably the most relevant measure of parasite diversity for PM studies. It is possible that the high number of *var2csa* sequence types reported here is due in part to parasites having multiple *var2csa* genes. While haploid in the human host, recent work has demonstrated that some isolates of *P.falciparum* contain more than one copy of *var2csa* [24], which may provide a selective advantage during the course of infection in pregnant women [25]. Furthermore, Brolin et.al. [26] has provided compelling evidence that both *var2csa* genes are transcribed simultaneously, suggesting *var2csa* expression in not a mutually exclusive process. To assess the number of unique genotypes within the Kenyan samples with a more conventional technique, we utilized a microsatellite analysis using seven markers (TA1, poly a, PfPK2, TA109, C2M34, C3M69). Our results indicate that the multiplicity of infection (MOI) ranged between 1–4 unique genotypes (**Table 2**). Whether due to greater polymorphism in *var2csa* than in the microsatellite markers, or to the potential for more than one copy of the gene to be present in a single genome, our results show 2–4 times more genetic complexity at the *var2csa* locus relative to loci tested in the microsatellite analysis. It is interesting to compare these findings with other studies conducted to assess MOI during pregnancy in Africa. Some of these studies used gel based PCR-RFLP analysis methods, and reported MOIs ranging from 1.5 to 4 [27], [28]. Another study used a quantitative Gene scan method and found a MOI of 5.6, suggesting that more sensitive methods are likely to detect higher MOIs [29]. The studies conflicted on the relationship between gravidity and MOI, with one study finding a negative correlation [29], and another finding no association [27]. Three other studies, two in Gabon [30], [31] and one in Ghana [32], also reported relatively low MOI (range of mean MOI 1.5–3). Again there was disagreement as to a correlation between gravidity and MOI as one study found decreased MOI with increasing gravidity [32] while the other found no correlation between MOI and age (an approximation for gravidity) [30]. Taken together, these studies found a variable MOI for placental parasite populations, but arrived at this conclusion via different techniques. While no correlation between genetic complexity within *var2csa* and gravidity was observed in the samples tested here, such an association, whether due to a higher multiplicity of infection or the multicopy nature of the *var2csa* gene in some isolates, likely needs to be tested by deeper analysis of the degree of complexity of placental infections.

**Table 1:** Summary of patient gravidity, total number of DBL3X sequences obtained, the number of clones sequenced and among these the number that were unique, and the nucleotide diversity.

Sample	Gravidity	Parasitemia Per 300 WBCs		Clones	Unique (nt/aa)	π	Pairwise Identity (%)
		Peripheral	Placental				
0593	P	24	3942	9	9/8	0.0047	99.5
0626	P	5	251	7	6/5	0.0401	94.4
0659	P	871	1745	8	7/6	0.0342	96.3
0696	P	798	1353	13	11/11	0.0447	93.1
0895	P	200	2534	11	2/2	-	99.9
0661	S	147	5429	10	9/9	0.0419	93.0
0786	S	563	4097	12	5/5	0.0366	95.8
0551	M	18	4	8	2/2	-	99.0
0608	M	650	929	9	9/9	0.0346	95.1
0694	M	242	325	11	9/9	0.0199	95.3
0833	M	3	15	9	9/9	0.0539	94.1
0855	M	3	72	1	1/1	-	-
	Totals:			108	**79/76**		

The value of π denotes the observed average pairwise nucleotide diversity.

**Table 2:** Summary of multi-locus genotyping using seven different markers (TA1, poly a, PfPK2, TA109, 2490, C2M34, and C3M69). DNW denotes loci for which there was no amplification after two attempts.

Chromosome		6	4	12	6	10	2	3
Locus		TA1	Polyα	PfPK2	TA109	2490	C2M34	C3M69
Sample ID	Genotypes	1	3	7	9	12	313	383
M0551	2	181	DNW	DNW	148 & 160	80	DNW	DNW
P0593	2	74 & 199	154	160	164 & 175	83	223	DNW
M0608	4	169 & 180	141, 144 & 151	162, 165, 169 & 184	140, 150 & 164	80 & 83	230	123
P0626	2	181	151 & 173	169 & 172	149 & 160	83	223 & 234	143 & 153
P0659	3	166	151 & 170	DNW	164, 167 & 175	83	DNW	DNW
S0661	4	159	137 & 170	163, 166, 169 & 184	146, 157, 163 & 176	80 & 83	DNW	123
P0696	1	DNW	170	163	200	80	255	135
S0786	4	175	147, 176 & 179	172 & 175	160, 163, 172 & 175	80 & 83	221, 230 & 234	149
M0833	3	74, 165 & 171	164	160 & 162	160, 163 & 175	83	223 & 232	118
P0895	2	166	167	165	167 & 178	86	243	140

**Figure 1:**
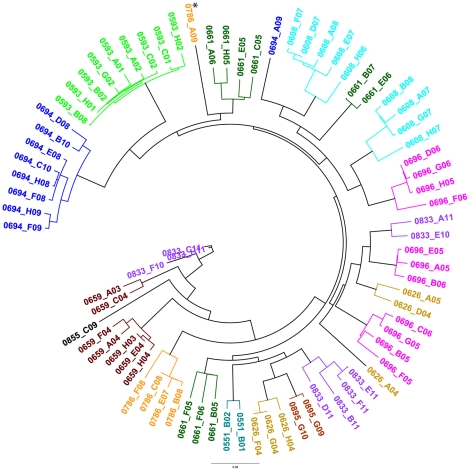
VAR2CSA in placental parasites shows unprecendented diversity with no interindividual clustering. Maxiumum Liklihood Tree of 79 Unique DBL3X Sequences. Sequences were aligned by translation. Consensus tree shown, with 50% cutoff. Taxa colored by placental sample. Sequences from the same patient sample tend to group together in one to three clades with the exception of 0786-A09, denoted by an asterisk.

Using these data, the 79 unique DBL3x sequences were further characterized by constructing a maximum likelihood tree (**Figure 1**). One interesting feature of the tree is that sequences from the same patient sample tend to group together in one to three clades. With the exception of one sequence, 0786-A09 (denoted with an asterisk), no sequences from one patient grouped in a clade with those of another patient. This result is in conflict with a similar analysis of 43 DBL3x sequences from 24 Senegalese placental samples by Dahlback et al. [33], who reported that sequences from different patient samples cluster together in distinct clades. Although it is difficult to know the precise reason for this discrepancy, the observed differences are likely due to the differences in study sample populations, transmission rates and the time span over which the samples were collected. In particular, one potential interpretation of this sequence grouping by patient among Kenyan samples is that the women came from different villages separated enough geographically to have distinct parasite populations, although no data were available to directly address this possibility.

**Figure 2:**
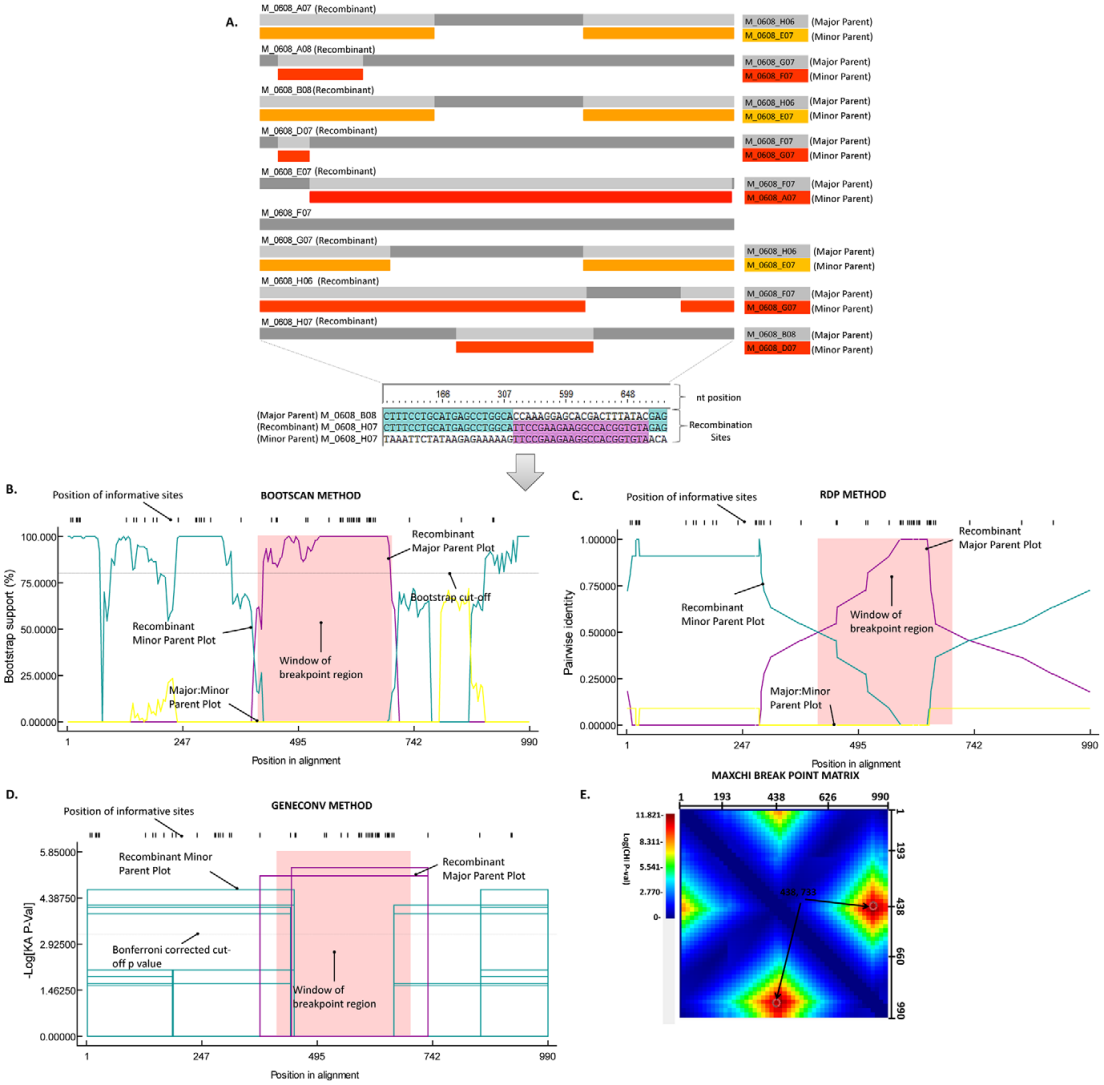
Identification of recombination events in a single patient and general protocol used for identifying recombination in all patient samples using RDP4. (A) RDP4 generated recombination schematic indicating evidence of intra-segmental recombination. Regions of recombination events, and corresponding insertions from donor sequences are depicted. Significant sites including nucleotide position(s) involved in recombination are shown for the last recombinant (M_0608_H07). Significant nucleotide positions involved in recombination were checked in a similar fashion for all other recombinants. Recombination events were only considered if at least three recombination analysis methods were in agreement with the obtained results, a *p-*value of 0.05 or better was obtained, and the consensus recombination score was above the recommended 0.60 value. An example for recombinant M_0608_HO7 showing recombination signals identified by the (B) BOOTSCAN method, (C) RDP method, and (D) GENECONV method are shown. Lastly, the MAXCHI matrices tool when necessary was used to determine the optimal locations of breakpoint pairs, shown in (E). These graphically represent the probabilities of all potential breakpoint pairs that have the best associated *p-*values displayed by the color key beside the matrix. Dark red peaks indicate the most probable positions of breakpoint pairs, for recombinant M_0608_HO7 these were at nucleotide positions 438 and 733.

Diversification within the *var* gene family has been associated with segmental gene recombination and gene conversion events [34], [35]. It has been recently demonstrated that *P. falciparum* has a relatively high recombination rate (RR) [36], including evidence of segmental gene recombination within *var2csa* alleles [37]. To identify whether the Kenyan samples diversified by novel amino acid mutations and/or segmental recombination, we analyzed the *var2csa* sequences using the Recombination Detection Program 4 (RDP4) [38] and by sequence alignment comparisons (**Figure S1**). The method and guidelines used in RDP4 for identifying recombination events at the global level (all sequences) and within the individual samples (same patient clusters) is shown in **Figure 2**. The data herein (**Figure 2**) represent analysis of recombination breakpoints related to the sequence of the transferred fragment (major parent sequence) and sequence closely related to the transferred fragment (minor parent) including the resulting recombinant, shown in **Table S2**. Considering all samples, the results of the RDP4 analysis revealed 14 unique recombination events, including evidence of recombination between sequences from different patients. Interestingly, analysis of sequences from individual patients suggests that more recombination is found at this level. As mentioned above, this could be due to geographic separation of patients, with each patient sample set reflecting recombination and diversification events occurring in an isolated locale. Although patient data collection did not include location of residence, it would be very interesting to assess this in future studies.

### Selective pressure in the DBL3X domain shows high sequence diversity and positive selection

**Figure 3:**
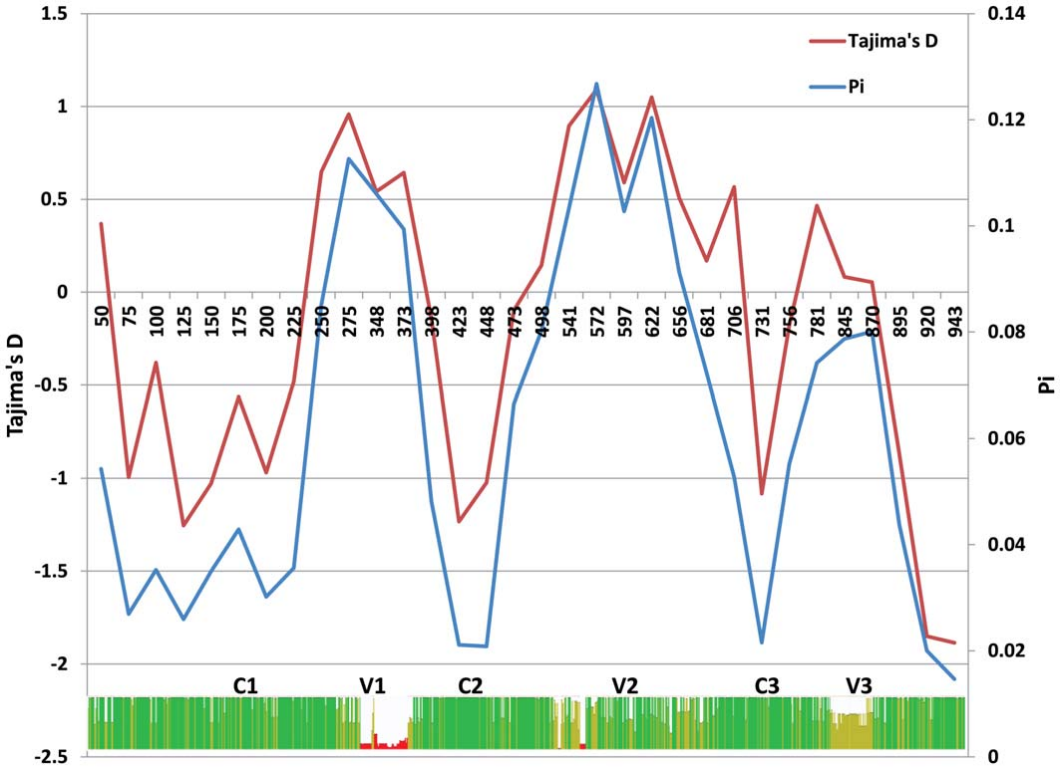
Evidence for selective pressure in regions of the DBL3X domain showing the highest sequence diversity. Overview of sequence diversity among DBL3X of Primigravidae and Multigravidae. The average pairwise similarity (π) and Tajima's D for the aligned sequences, over an 870 nucleotide window including Conserved (C1–4) and variable (V1–3) regions are shown.

Analysis of sequence polymorphism at the population level is an effective tool for detecting influence of continuing selection. Sites experiencing selection show either a decrease or increase in the density of polymorphism. To further investigate DBL3X polymorphism and regions under selective pressure, the pairwise diversity across the sequences was calculated, and Tajima's Neutrality Test was applied to reveal selection hotspots (see Materials and Methods for details). The observed average pairwise diversity (л) values for primigravidae (0.0582) and multigravidae (0.0507) are similar (**Table 3**) suggesting that there is no influence of gravidity or, by extension, host immunity on the overall diversity of *var2csa* in placental infections. Still, this observed pairwise diversity is considerably higher than observed for other malarial antigens such as *eba-175* (π = 0.00366), *ebl-1* (π = 0.00321), and *eba-140* (π = 0.001) [39], [40]. This highlights that while *var2csa* is a conserved member of the *var* gene family, it is highly polymorphic when compared to other malarial antigens considered to be viable vaccine candidate antigens. When π is calculated for each position in an alignment of DBL3X sequences it is clear that the diversity in this domain is primarily found in the first and second variable regions (V1 and V2), and to a lesser degree the third variable region (V3) (**Figure 3**). While Tajima's D is not >1 when calculated over the entire DBL3X domain (**Table 3**), certain regions of this domain do show evidence of positive selection; that is, Tajima's D (departure from neutrality) is >1 for those regions exhibiting the highest degree of pairwise diversity (**Figure 2**). This is not surprising as positive selection will favor escape mutations. These results are in agreement with other studies that have examined diversity in DBL3X [33], [37], and provide good evidence that this domain is a target of immune selection.

**Table 3:** Results from Tajima's Neutrality Test.

	m	S	p_s_	Θ	π	D
**Primigravid**	35	172	0.195011	0.047353	0.058227	0.866659
**Multigravid**	30	178	0.201130	0.050769	0.059910	0.695683

The Tajima test statistic [1] was estimated using MEGA4 [2]. All positions containing gaps and missing data were eliminated from the dataset (Complete deletion option). The abbreviations used are as follows: m = number of sites, S = Number of segregating sites, p_s_ = S/m, Θ = p_s_/a_1_, and π = nucleotide diversity. D is the Tajima test statistic.

### Novel motifs identified in Kenyan samples

Amino acid sequence alignments are frequently used in the analysis of protein structure, function and evolutionary relationships. Analysis of positional amino acid conservation of sequences can aid in identifying critical motifs and structurally important residues. Dahlback et al [33] reported that the sequence motif EIEKD at amino acid positions 1423–1427 in V2 was overrepresented in primigravidae, while EIERE/EIEGE/GIERE and GIEGE were predominantly found in multigravid women. Four of these motifs, GIERE, EIEKD, EIERE, and GIEGE, were present in the Kenyan DBL3X sequences, but the motif EIEGE was not observed. GIEGE was somewhat overrepresented in multigravidae, with 69% versus 31% in primigravidae. The motif EIEKD and EIERE/GIERE were not conclusively gravidity-associated in these samples. Finally, unique to the Kenyan samples, the motif TKQN at amino acid positions 1401–1404 in V2 was mainly found in primigravidae and TKTK/PQQK in multigravidae (**Table 4**). These motifs, therefore, show statistically significant association with host immune status, using the Fisher's exact test to compare the novel motifs in multigravidae and primigravidae. Overall, these results suggest that the Senegalese and Kenyan parasite populations are separated enough geographically to have developed distinct motifs that may provide a biological advantage based upon gravidity. It is also possible that host genotypic differences in immune-relevant function have provided unique selective pressures on local parasites, thus reflecting geographically unique host/parasite co-evolution.

**Table 4:** Novel motifs identified in Kenyan samples are associated with host immune status (gravidity).

Sequence Motif	Primigravid	Multigravid
EIKD	59%	41%
EIERE/GIERE	41%	59%
GIEGE	31%	69%
TKQN*	28%	72%
TKTK*	80%	20%
PQQK*	75%	25%

Motifs TKTK/PQQK are predominately found in primigravidae and TKQN in multigravidae. Sequence motifs (highlighted in grey) identified in Dahlback et al. [33] show no strong parity association in Kenyan samples. * *P*<0.001, by c^2^ test.

### Lowest sequence conservation in the DBL3X domain observed near the predicted binding pocket of CSA and the critical amino acid residue arginine implicated in binding to placental CSA is not absolutely conserved

**Figure 4:**
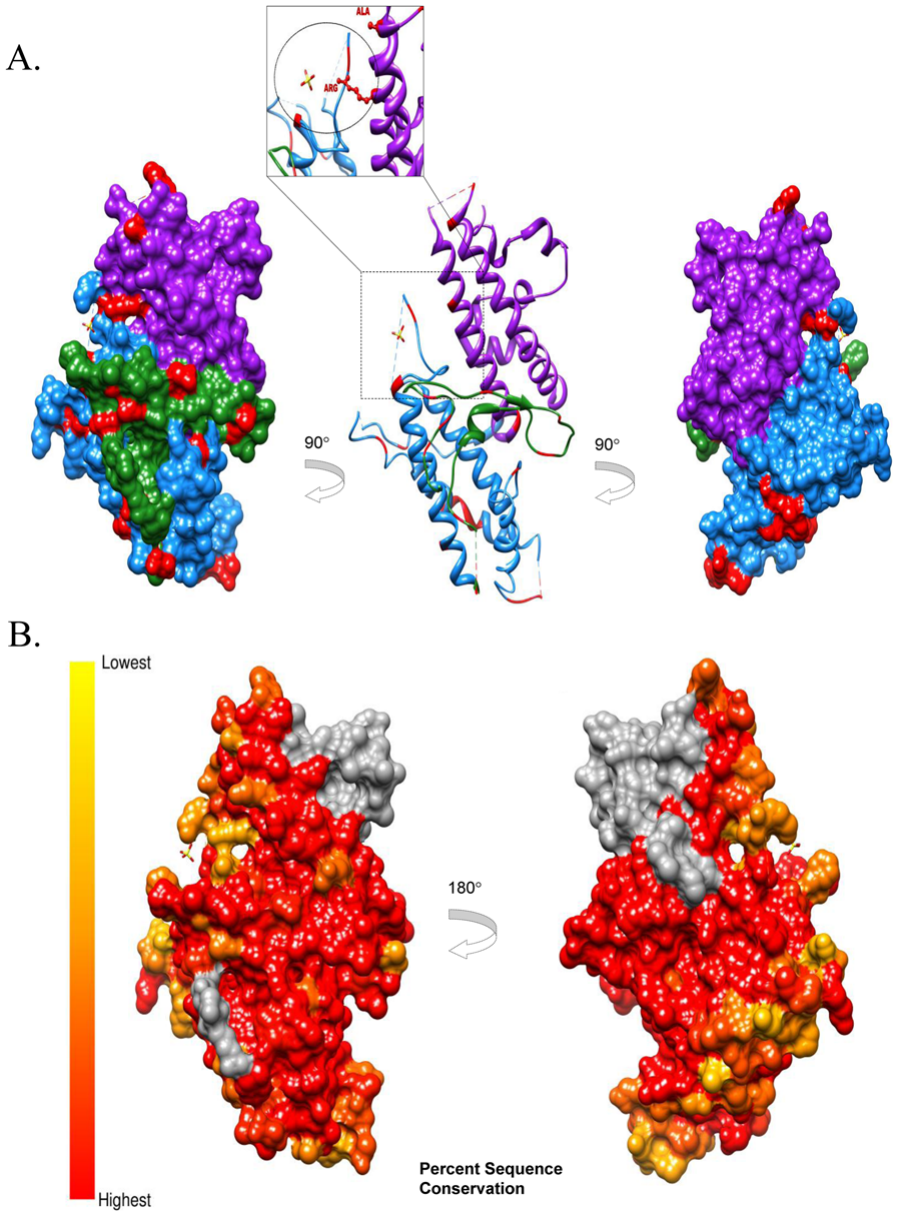
Toggling Amino Acid Regions and Amino Acid Conservation of VAR2CSA DBL3X Domain. (A) DBL3X surface and ribbon representation of subdomain I (green), subdomain II (dodger blue), subdomain III (magenta) and toggling regions (red). CSA sulfate ion is shown in the proposed binding pocket. A proposed binding residue of CSA, arginine1467 was identified as a toggling residue (shown by blown up image). (B) Analysis of Amino Acid Conservation of VAR2CSA DBL3X domain. Percent sequence conservation from highest 100% (red) to lowest 40% (yellow) based on an alignment of 79 DBL3X sequences isolated from twelve Kenyan placental blood samples is shown on a DBL3X surface based model. Dotted circles indicate the predicted binding pocket region for CSA, shown with an SO4 molecule. Insufficient sequence data for analysis is depicted in grey.

Conservation analysis has proven to be a powerful indicator of functional importance and has been used to determine residues implicated in ligand binding [41], [42], protein-protein interactions [43], [44], and functional specificity [44], [45]. Although many computation methods are available for determining the functional importance of residues, it has been found that conservation is one of the most powerful attributes in these applications [46]. Mapping this information on a 3D structure can help visualize potential functional surfaces (e.g. such as those exposed to host humoral immunity). Toward this end, the protein structure of DBL3X (Protein Data Bank: 3CPZ, [47]) was used as a template for structural modeling of the 79 unique DBL3X sequences and analysis of sequence conservation performed using the UCSF Chimera molecular analysis program [48] (see Materials and Methods for details). The structure of the DBL3X domain is made up of three subdomains [47], [49]. Subdomain I (S1) (**Figure 4A** **, green**), primarily forms a large loop with little secondary structure. Subdomain II (S2) (**Figure 4A** **, dodger blue**), comprises four helices connected by loops. Subdomain III (S3) (**Figure 4A** **, magenta**), is comprised of two anti-parallel helices. Conservation analysis of DBL3X reveals that lowest sequence conservation is focused around the predicted binding pocket, suggesting this region may be under immune pressure (**Figure 4B**). S3 has recently been identified as a minimal CSA binding region and mutations of residues within this subdomain result in substantial reduction of binding to placental CSA [50]. Of note, it has recently been shown that expression constructs containing the N-terminal domains [18], including the DBL3X domain [20], provide high specificity binding comparable to full-length VAR2CSA. Furthermore, as suggested by Bigley et al., only sequences between the NTS and the DBL3X appear to induce inhibitory antibodies [51]. This would suggest that DBL3X in the presence of other N-terminal domains plays a critical role in the native protein structure.

**Figure 5:**
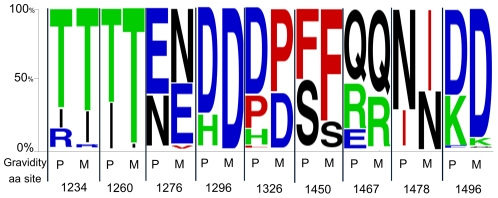
Toggling amino acids with gravidity-associated preferences. A total of nine sites were identified as varying significant between primigravidae (P) and multigravidae (M), shown here in relative proportion of toggling amino acids at each site (see Table S1 for details and other toggling sites). The chi-square test (or Fisher's exact test when appropriate) was used; all sites, *P*<0.05.

Epitopes exposed on the surface of native VAR2CSA may therefore be accessible to immunoglobulins and, as a result, be under strong selective pressure for escape mutations. While host immune responses exert strong selective pressures resulting in amino acid positions that exhibit a pattern of escape mutations, there can be functional consequences to these changes which drive select for reversion mutations in a naïve host. Such mutations within or flanking functionally important epitopes can have two effects: (1) immune evasion through mutation from a wild type sequence driven by selective pressure and (2) changing ligand binding for potentially improved or reduced affinity. This process of mutation and reversion has been termed “toggling” in a recent study by Delport et al. [52] who used a probabilistic model of protein sequences in HIV-1 to demonstrate that there is a large number of sites evolving under selective pressure, but which also exhibit low sequence diversity. These toggling sites, typically, were found to switch between just 2 or 3 amino acids when comparing different viral isolates. That observation prompted an evaluation of the Kenyan samples to determine the extent to which toggling between wild type and escape amino acids is evident and whether or not such toggling sites can be used to identify specific regions of DBL3X that are subject to immune pressure and, therefore, likely to encode functionally important amino acids. Toward this end, the 79 unique DBL3x sequences were submitted for toggling analysis to the Datamonkey server (see Materials and Methods for details) and the results mapped to the protein structure of DBL3X as above. This analysis yielded a total of 27 toggling amino acids, primarily found on domains S1 and S2 (Figure 3). It is interesting to note that an arginine residue (Arg^1467^) on S3, previously implicated in binding to the placental receptor CSA [15], [47], [50], toggles between arginine (R), glutamine (Q), and glutamic acid (E). A recent study by Khunrae et al. [15] suggests that CSA binding is mediated by the positively charged patch in the binding pocket. This would imply that the toggling between a positive (R) – negative (Q) – positive (E) charge may modulate the binding specificity of the N-terminal domains to placental CSA and reflects the pressure on the parasite to forego efficient binding in the face of acquired immune clearance mechanisms. Of note, a single amino acid substitution in *P. vivax* Duffy-binding protein (PvDBP) was found to dramatically alter both its binding affinity and antigenic character [53]. To characterize the individual sites relative to immune status, relative proportions of toggling amino acids at each site in primigravidae and multigravidae were compared (**Table S1)**. As shown in **Figure 5**, nine of the 27 toggling sites were found to toggle differentially as a function of gravidity. Since host immune responses exert strong selective pressures favoring mutations that prevent immune recognition, these toggling sites (especially Arginine^1467^ on S3) may represent important epitopes that, when targeted by host antibody responses, force the parasite to choose between efficient binding to CSA and mutation to facilitate immune evasion. A consequence of such mutations may be decreased specificity for binding of infected erythrocytes to placental CSA. This hypothesis would be intriguing to test in future studies and extend the toggling analysis to the NTS-DBL2X domain region.

### Concluding Remarks

Numerous studies have addressed sequence polymorphism in *var2csa*. After performing similar alignment, tree and structural analyses as previous studies, the present results reveal agreements and discrepancies with the available published data. Notably, the present analysis revealed a high level of parasite genetic complexity within individual patient samples at the *var2csa* locus. Given that an average of nine clones per sample were examined, the true genetic complexity in each sample is likely to be considerably higher and will require additional investigation.

While it is possible that the multicopy nature of the *var2csa* gene in some isolates may account for some of the observed complexity, we provide evidence that intra-segmental recombination and amino acid mutations driven by immune pressure appear to play a critical role in generating the observed diversity. While we examined a limited set of samples, our results reveal several statistically significant gravidity-associated motifs; intriguingly, the patterns and identity of the motifs differ from those previously reported in Senegalese samples [33]. This discrepancy may reflect geographically unique host/parasite co-evolution, but will require analysis of additional samples from distinct greographic regions to confirm this hypothesis. An important advance in this study was the application of *in silico* methods to identify recombination events and functionally important positions that toggle between a limited set of amino acids and may impact binding to placental CSA. Overall, the nine toggling positions that vary significantly between primigravidae and multigravidae in terms of which amino acid is preferred provide indirect evidence that acquired immunity to CSA-binding parasites that is elicited by repeated exposure to PM selects for parasites with different sequences than those used by parasites occupying an immunologically naïve environment. It is of particular interest that most of these cases reveal a tendency toward homogeneity in multigravidae. Further study will be required to evaluate the extent to which the toggling in the full NTS-DBL3X region is influenced by immune responses and affects efficiency of binding to CSA.

As previously observed, detailed sequence analysis coupled with 3D modeling of DBL3X reveals that this domain is very complex. However, the genetic complexity and multiplicity of infection identified in this study is likely to have underestimated the true diversity of infection in pregnant women. The toggling amino acid sites identified in this study were limited to the DBL3X domain; it would be interesting to test the hypothesis that successful parasites have limited choices for some residues within VAR2CSA, especially the N-terminal region. Our data are theoretically consistent with a dynamic host/parasite relationship in which immune pressure in a multigravid host selects for escape mutations that later revert back under selection for binding function in a naïve, primigravid host. Should these sites be confirmed as targets for protective antibodies, then such information could be used in the design of a VAR2CSA vaccine construct that targets different serological variants of these regions.

## Materials and Methods

### Sample Collection

The samples used in this study were derived from a project designed to assess gravidity-dependent acquisition of cell-mediated immunity to PM as previously described [54]. In brief, samples were collected from women delivering at Siaya District Hospital in western Kenya. This is an area with stable malaria transmission all year round with reported entomological inoculation rates (EIR) estimated as high as 31 infective bites/person/year [55]. Informed consent was obtained from potential clients in agreement with Kenya Medical Research Institute Ethical Review Committee, the Institutional Review Boards of the University of Georgia, the Centers for Disease Control and Prevention, and the National Institutes of Health. All participants provided informed, written consent under the auspices of these approved protocols. Immediately post-delivery, placentas were collected and maternal placental blood obtained either by the prick method [56] or perfusion [57] under aseptic conditions. Placental parasitemia was calculated by counting the number of parasites on a Giemsa-stained thick smear of maternal placental prick blood and is reported as parasitemia per 300 white blood cells. Each of the collected samples is well defined by clinical data (age, gravidity, birth weight). Samples for this study were collected over a time span of 1.5 years.

### Amplification and Sequencing

DNA was extracted from placental blood samples using the illustra™ blood genomicPrep™ Mini Spin Kit (GE Healthcare, Little Chalfont, Buckinghamshire, UK) according to manufacturer's instructions. Primers to conserved regions of the 5′ (BacdiDBL3F - CACCATGAATTATATTCGTGGGTGTCAA) and 3′ (BacdiDBL3R – ATTTGCTGATATACATTCAGG) boundaries of the var2csa DBL3X domain were designed from an alignment of 5 *var2csa* sequences. For each sample, the DBL3Xdomain was amplified using the proof reading ExTaq (Takara Bio Inc.) and cloned via the TOPO reaction into the pENTR/D-TOPO vector (Invitrogen, Carlsbad, CA). For each sample a minimum of three independent amplification and TOPO reactions were performed. The samples were chosen to include a range of gravidities with 5 primigravid, 2 secundigravid and 5 multigravid women. The TOPO reactions were transformed into competent *E. coli* and plated to obtain cloned plasmids. Colonies containing plasmids with a DBL3X insert were identified via colony PCR. Multiple clones were obtained for each sample and submitted for sequencing. A total of 6 overlapping reads were obtained for each sequence using vector primers and four sequence specific primers (DBL3F.2 TGATTATAAGAATATGATTTTGGGTAC, DBL3R.2 TTTATATCTTCTTGTAATTTTCCAATG, DBL3F.3 GAATACCACGATAAAGGTACAGC, DBL3R.3 CCACGAAACGAACTGATCCTC).

Following sequencing, all chromatograms were checked for accuracy and quality, and reads in both directions were assembled into full length contigs for each clone using the Geneious software package (Biomatters Ltd, Auckland NZ). We obtained between 7 and 13 complete DBL3X sequences per sample, with the exception of one sample for which a single sequence was obtained. As a control, the amplification of DBL3Xregion from the *var2csa* of isolate FCR3/IT4 was used. All three independent FCR3 sequences that were obtained perfectly matched the reported sequence (GenBank accession number AY372123), suggesting a very low error rate. From the 10 placental blood samples a total of 108 sequences were obtained. Taking a conservative approach, sequences were only considered unique if they differed by more than 2 base pairs. All unique sequences, 79 in all, were submitted to Genbank under accession numbers JN615483–JN615561.

### Phylogenetic Analysis and Neutrality Test

To characterize the 79 unique DBL3X sequences, along with the FCR3/IT4 sequence, alignments were done in Geneious Pro using the ClustalW algorithm and phylogenetic analysis was performed in MEGA [58]. Tajima's D test as implemented in both MEGA and DnaSP [59] was used to test 35 multigravidae and 30 primigravidae DBL3X sequences for departure from neutrality in the nucleotide frequency distributions (secundigravidae were excluded from analysis since only 15 sequences from 2 individuals were available). This is determined by divergence in the values of *π* (observed average pairwise nucleotide diversity) and *θ* (expected nucleotide diversity under neutrality derived from the number of segregating sites, *S*). Elevated *π* and positive values to D would confer a balancing selection of nucleotide sites that would be maintained at intermediate frequencies.

### Multilocus Genotyping

Twelve DNA samples were assayed for seven putatively neutral microsatellites as described previously [60]. The neutral markers used were TA1 found on chromosome 6; poly α, chromosome 4; PfPK2, chromosome 12; TA109, chromosome 6 and 2490, chromosome 10; these neutral markers were previously described and used in various studies in South America [51], [61], [62], [63]. The final two neutral microsatellite markers used were C2M34, chromosome 2 and C3M69, chromosome 3 [64]. Briefly, the amplification products were labeled with fluorescent dyes (HEX or FAM) and assayed for size on an Applied Biosystems 3130xl sequencer (Applied Biosystems, Foster City, CA). The fragments were then scored with GeneMapper software v.3.7 (Applied Biosystems, Foster City, CA) using default microsatellite settings, whereby bands smaller than 1000 relative fluorescence units (rfu) were defined as background. Samples for which we obtained no amplification in some loci or for which we obtained multiple alleles at certain loci were re-analyzed to complete the analysis.

### 3D Structure Modeling, Percent Sequence Conservation

The protein structure of DBL3X (Protein Data Bank: 3CPZ, [47]) was used as a template for structure modeling. Structural visualizations and labeling was done using the UCSF Chimera molecular analysis program [48]. Briefly, the 79 unique DBL3x sequences including the FCR3/IT4 sequence was aligned using Chimera's Multialign View feature and sequence conservation was calculated using the mavPercentConservation method. The mavPercentConservation method is based on the AL2CO algorithm. The algorithm of AL2CO program performs calculations in two steps. First, amino acid frequencies at each position are estimated and then the conservation index is calculated from these frequencies. The results were then mapped to the protein structure of DBL3x (Protein Data Bank: 3CPZ, [47]) using the following color parameters: lowest (40%) and highest (100%) sequence conservation are represented in yellow and red, respectively. Insufficient data (shown in grey) indicates amino acid locations that were missing from our sequences.

### Toggling Amino Acid Analysis and 3D Structure Mapping

Toggling amino acid analysis was performed on 79 sequences obtained from 12 placental blood samples using Datamonkey: a suite of phylogenetic analysis tools for evolutionary biology [65]. Briefly, the sequences were uploaded on the Datamonkey server and analysis was performed using the following four steps: First, a nucleotide model was fitted to the DBL3X sequences using maximum likelihood to obtain branch lengths and substitution rates. Second, the data were fitted to obtain a global ω = dN/dS ratio. Next, codon ancestral sequences were constructed site by site using maximum likelihood. Lastly, a test of escape and reversion from/to the wild type was conducted and results reported with the following summary statistics: the likelihood ratio test statistic, *P*-value, toggling rate and the proportion of time each site “spends” in the wild-type, single-step and multiple-step escape amino acid states. Each of the toggling amino acids was then mapped to the template sequence of the protein structure of DBL3X (Protein Data Bank: 3CPZ, [47]) and analyzed using the UCSF Chimera molecular analysis program.

### Statistical Analysis

The chi-square test (or Fisher's exact test when appropriate) was used to compare novel motifs and toggling amino acid proportions between multigravidae and primigravidae. Statistically significant (*P*<0.05) sites were mapped using WebLogo [66].

## Supporting Information

Figure S1 **Amino acid alignment of 79 var2csa DBL3x domains.** Individual subdomains are denoted by arrows above alignment. Toggling sites are marked by a “T” above the relevant amino acid site. Motifs identified in this study and in Dahlback at al. [33] are denoted by black boxes. Amino acid residues are colored according to conservation.
(PDF)

Table S1 **Relative proportions of toggling amino acids at each amino acid position.** Amino acids found to toggle were stratified by gravidity. Relative proportions of occupying amino acids at each respective site within each group are represented as a percentage. Nine sites (shown in grey and denoted by a asterisk) were found to differ statistically significantly (c2, *P*<0.05) between the gravidity groups.
(PDF)

Table S2 **Summary table of recombination analysis via RDP4** [**38**] **at a global level (all sequences) and individual samples (same patient clusters).** Data shows recombination breakpoints related to the sequence of the transferred fragment (major parent) and sequence closely related to transferred fragment (minor parent) including resulting recombinant. For statistical analysis the Chi-square statistical test in RDP4 was used.
(PDF)

## References

[1] Dellicour S, Tatem AJ, Guerra CA, Snow RW, ter Kuile FO (2010). Quantifying the number of pregnancies at risk of malaria in 2007: a demographic study.. PLoS Med.

[2] Brabin BJ, Romagosa C, Abdelgalil S, Menendez C, Verhoeff FH (2004). The sick placenta-the role of malaria.. Placenta.

[3] Salanti A, Staalsoe T, Lavstsen T, Jensen AT, Sowa MP (2003). Selective upregulation of a single distinctly structured var gene in chondroitin sulphate A-adhering Plasmodium falciparum involved in pregnancy-associated malaria.. Mol Microbiol.

[4] Reeder JC, Cowman AF, Davern KM, Beeson JG, Thompson JK (1999). The adhesion of Plasmodium falciparum-infected erythrocytes to chondroitin sulfate A is mediated by P. falciparum erythrocyte membrane protein 1.. Proc Natl Acad Sci U S A.

[5] Fried M, Duffy PE (1996). Adherence of Plasmodium falciparum to chondroitin sulfate A in the human placenta.. Science.

[6] Tuikue Ndam NG, Salanti A, Bertin G, Dahlback M, Fievet N (2005). High level of var2csa transcription by Plasmodium falciparum isolated from the placenta.. J Infect Dis.

[7] Viebig NK, Gamain B, Scheidig C, Lepolard C, Przyborski J (2005). A single member of the Plasmodium falciparum var multigene family determines cytoadhesion to the placental receptor chondroitin sulphate A.. EMBO Rep.

[8] Ricke CH, Staalsoe T, Koram K, Akanmori BD, Riley EM (2000). Plasma antibodies from malaria-exposed pregnant women recognize variant surface antigens on Plasmodium falciparum-infected erythrocytes in a parity-dependent manner and block parasite adhesion to chondroitin sulfate A.. J Immunol.

[9] Staalsoe T, Shulman CE, Bulmer JN, Kawuondo K, Marsh K (2004). Variant surface antigen-specific IgG and protection against clinical consequences of pregnancy-associated Plasmodium falciparum malaria.. Lancet.

[10] Maubert B, Fievet N, Tami G, Cot M, Boudin C (1999). Development of antibodies against chondroitin sulfate A-adherent Plasmodium falciparum in pregnant women.. Infect Immun.

[11] Fried M, Nosten F, Brockman A, Brabin BJ, Duffy PE (1998). Maternal antibodies block malaria.. Nature.

[12] Rowe JA, Kyes SA, Rogerson SJ, Babiker HA, Raza A (2002). Identification of a conserved Plasmodium falciparum var gene implicated in malaria in pregnancy.. J Infect Dis.

[13] Salanti A, Dahlback M, Turner L, Nielsen MA, Barfod L (2004). Evidence for the involvement of VAR2CSA in pregnancy-associated malaria.. J Exp Med.

[14] Duffy MF, Maier AG, Byrne TJ, Marty AJ, Elliott SR (2006). VAR2CSA is the principal ligand for chondroitin sulfate A in two allogeneic isolates of Plasmodium falciparum.. Mol Biochem Parasitol.

[15] Khunrae P, Philip JM, Bull DR, Higgins MK (2009). Structural comparison of two CSPG-binding DBL domains from the VAR2CSA protein important in malaria during pregnancy.. J Mol Biol.

[16] Gamain B, Trimnell AR, Scheidig C, Scherf A, Miller LH (2005). Identification of multiple chondroitin sulfate A (CSA)-binding domains in the var2CSA gene transcribed in CSA-binding parasites.. J Infect Dis.

[17] Avril M, Gamain B, Lepolard C, Viaud N, Scherf A (2006). Characterization of anti-var2CSA-PfEMP1 cytoadhesion inhibitory mouse monoclonal antibodies.. Microbes Infect.

[18] Srivastava A, Gangnard S, Round A, Dechavanne S, Juillerat A (2010). Full-length extracellular region of the var2CSA variant of PfEMP1 is required for specific, high-affinity binding to CSA.. Proc Natl Acad Sci U S A.

[19] Srivastava A, Gangnard S, Dechavanne S, Amirat F, Lewit Bentley A (2011). Var2CSA Minimal CSA Binding Region Is Located within the N-Terminal Region.. PLoS One.

[20] Dahlback M, Jorgensen LM, Nielsen MA, Clausen TM, Ditlev SB (2011). The chondroitin sulfate A-binding site of the VAR2CSA protein involves multiple N-terminal domains.. J Biol Chem.

[21] Gamain B, Smith JD, Viebig NK, Gysin J, Scherf A (2007). Pregnancy-associated malaria: parasite binding, natural immunity and vaccine development.. Int J Parasitol.

[22] Hommel M, Elliott SR, Soma V, Kelly G, Fowkes FJ (2010). Evaluation of the antigenic diversity of placenta-binding Plasmodium falciparum variants and the antibody repertoire among pregnant women.. Infect Immun.

[23] Barfod L, Bernasconi NL, Dahlback M, Jarrossay D, Andersen PH (2007). Human pregnancy-associated malaria-specific B cells target polymorphic, conformational epitopes in VAR2CSA.. Mol Microbiol.

[24] Sander AF, Salanti A, Lavstsen T, Nielsen MA, Magistrado P (2009). Multiple var2csa-type PfEMP1 genes located at different chromosomal loci occur in many Plasmodium falciparum isolates.. PLoS One.

[25] Sander AF, Salanti A, Lavstsen T, Nielsen MA, Theander TG (2011). Positive selection of Plasmodium falciparum parasites with multiple var2csa-type PfEMP1 genes during the course of infection in pregnant women.. J Infect Dis.

[26] Brolin KJ, Ribacke U, Nilsson S, Ankarklev J, Moll K (2009). Simultaneous transcription of duplicated var2csa gene copies in individual Plasmodium falciparum parasites.. Genome Biol.

[27] Schleiermacher D, Le Hesran JY, Ndiaye JL, Perraut R, Gaye A (2002). Hidden Plasmodium falciparum parasites in human infections: different genotype distribution in the peripheral circulation and in the placenta.. Infect Genet Evol.

[28] Leke RF, Bioga JD, Zhou J, Fouda GG, Leke RJ (2010). Longitudinal studies of Plasmodium falciparum malaria in pregnant women living in a rural Cameroonian village with high perennial transmission.. Am J Trop Med Hyg.

[29] Schleiermacher D, Rogier C, Spiegel A, Tall A, Trape JF (2001). Increased multiplicity of Plasmodium falciparum infections and skewed distribution of individual msp1 and msp2 alleles during pregnancy in Ndiop, a Senegalese village with seasonal, mesoendemic malaria.. Am J Trop Med Hyg.

[30] Kassberger F, Birkenmaier A, Khattab A, Kremsner PG, Klinkert MQ (2002). PCR typing of Plasmodium falciparum in matched peripheral, placental and umbilical cord blood.. Parasitol Res.

[31] Mayengue PI, Rieth H, Khattab A, Issifou S, Kremsner PG (2004). Submicroscopic Plasmodium falciparum infections and multiplicity of infection in matched peripheral, placental and umbilical cord blood samples from Gabonese women.. Trop Med Int Health.

[32] Beck S, Mockenhaupt FP, Bienzle U, Eggelte TA, Thompson WN (2001). Multiplicity of Plasmodium falciparum infection in pregnancy.. Am J Trop Med Hyg.

[33] Dahlback M, Rask TS, Andersen PH, Nielsen MA, Ndam NT (2006). Epitope mapping and topographic analysis of VAR2CSA DBL3X involved in P. falciparum placental sequestration.. PLoS Pathog.

[34] Trimnell AR, Kraemer SM, Mukherjee S, Phippard DJ, Janes JH (2006). Global genetic diversity and evolution of var genes associated with placental and severe childhood malaria.. Mol Biochem Parasitol.

[35] Kraemer SM, Kyes SA, Aggarwal G, Springer AL, Nelson SO (2007). Patterns of gene recombination shape var gene repertoires in Plasmodium falciparum: comparisons of geographically diverse isolates.. BMC Genomics.

[36] Jiang H, Li N, Gopalan V, Zilversmit MM, Varma S (2011). High recombination rates and hotspots in a Plasmodium falciparum genetic cross.. Genome Biol.

[37] Bockhorst J, Lu F, Janes JH, Keebler J, Gamain B (2007). Structural polymorphism and diversifying selection on the pregnancy malaria vaccine candidate VAR2CSA.. Mol Biochem Parasitol.

[38] Martin DP, Lemey P, Lott M, Moulton V, Posada D (2010). RDP3: a flexible and fast computer program for analyzing recombination.. Bioinformatics.

[39] Baum J, Thomas AW, Conway DJ (2003). Evidence for diversifying selection on erythrocyte-binding antigens of Plasmodium falciparum and P. vivax.. Genetics.

[40] Drummond PB, Peterson DS (2005). An analysis of genetic diversity within the ligand domains of the Plasmodium falciparum ebl-1 gene.. Mol Biochem Parasitol.

[41] Liang S, Zhang C, Liu S, Zhou Y (2006). Protein binding site prediction using an empirical scoring function.. Nucleic Acids Res.

[42] Magliery TJ, Regan L (2005). Sequence variation in ligand binding sites in proteins.. BMC Bioinformatics.

[43] Guharoy M, Chakrabarti P (2005). Conservation and relative importance of residues across protein-protein interfaces.. Proc Natl Acad Sci U S A.

[44] Hannenhalli SS, Russell RB (2000). Analysis and prediction of functional sub-types from protein sequence alignments.. J Mol Biol.

[45] Lichtarge O, Bourne HR, Cohen FE (1996). An evolutionary trace method defines binding surfaces common to protein families.. J Mol Biol.

[46] Petrova NV, Wu CH (2006). Prediction of catalytic residues using Support Vector Machine with selected protein sequence and structural properties.. BMC Bioinformatics.

[47] Singh K, Gittis AG, Nguyen P, Gowda DC, Miller LH (2008). Structure of the DBL3x domain of pregnancy-associated malaria protein VAR2CSA complexed with chondroitin sulfate A.. Nat Struct Mol Biol.

[48] Pettersen EF, Goddard TD, Huang CC, Couch GS, Greenblatt DM (2004). UCSF Chimera–a visualization system for exploratory research and analysis.. J Comput Chem.

[49] Higgins MK (2008). The structure of a chondroitin sulfate-binding domain important in placental malaria.. J Biol Chem.

[50] Singh K, Gitti RK, Diouf A, Zhou H, Gowda DC (2010). Subdomain 3 of Plasmodium falciparum VAR2CSA DBL3x is identified as a minimal chondroitin sulfate A-binding region.. J Biol Chem.

[51] Bigey P, Gnidehou S, Doritchamou J, Quiviger M, Viwami F (2011). The NTS-DBL2X region of VAR2CSA induces cross-reactive antibodies that inhibit adhesion of several plasmodium falciparum isolates to chondroitin sulfate A.. J Infect Dis.

[52] Moore RM, Venugopalan CS, Sedrish SA, Holmes EP (1997). Role of endothelium and nitric oxide in the in vitro response of equine colonic venous rings to vasoconstrictor agents.. Am J Vet Res.

[53] McHenry AM, Barnes SJ, Ntumngia FB, King CL, Adams JH (2011). Determination of the Molecular Basis for a Limited Dimorphism, N417K, in the Plasmodium vivax Duffy-Binding Protein.. PLoS One.

[54] Perrault SD, Hajek J, Zhong K, Owino SO, Sichangi M (2009). Human immunodeficiency virus co-infection increases placental parasite density and transplacental malaria transmission in Western Kenya.. Am J Trop Med Hyg.

[55] Ndenga B, Githeko A, Omukunda E, Munyekenye G, Atieli H (2006). Population dynamics of malaria vectors in western Kenya highlands.. J Med Entomol.

[56] Othoro C, Moore JM, Wannemuehler K, Nahlen BL, Otieno J (2006). Evaluation of various methods of maternal placental blood collection for immunology studies.. Clin Vaccine Immunol.

[57] Moore JM, Nahlen B, Ofulla AV, Caba J, Ayisi J (1997). A simple perfusion technique for isolation of maternal intervillous blood mononuclear cells from human placentae.. J Immunol Methods.

[58] Tamura K, Peterson D, Peterson N, Stecher G, Nei M (2011). MEGA5: Molecular Evolutionary Genetics Analysis using Maximum Likelihood, Evolutionary Distance, and Maximum Parsimony Methods.. Mol Biol Evol.

[59] Librado P, Rozas J (2009). DnaSP v5: a software for comprehensive analysis of DNA polymorphism data.. Bioinformatics.

[60] Chamberlin A, Mitsuhashi Y, Bigley K, Bauer JE (2011). Unexpected depletion of plasma arachidonate and total protein in cats fed a low arachidonic acid diet due to peroxidation.. Br J Nutr.

[61] Dickinson RE, Griffin H, Bigley V, Reynard LN, Hussain R (2011). Exome sequencing identifies GATA-2 mutation as the cause of dendritic cell, monocyte, B and NK lymphoid deficiency.. Blood.

[62] Hoffmann EH, Ribolla PE, Ferreira MU (2003). Genetic relatedness of Plasmodium falciparum isolates and the origin of allelic diversity at the merozoite surface protein-1 (MSP-1) locus in Brazil and Vietnam.. Malar J.

[63] Machado RL, Povoa MM, Calvosa VS, Ferreira MU, Rossit AR (2004). Genetic structure of Plasmodium falciparum populations in the Brazilian Amazon region.. J Infect Dis.

[64] McCollum AM, Mueller K, Villegas L, Udhayakumar V, Escalante AA (2007). Common origin and fixation of Plasmodium falciparum dhfr and dhps mutations associated with sulfadoxine-pyrimethamine resistance in a low-transmission area in South America.. Antimicrob Agents Chemother.

[65] Delport W, Poon AF, Frost SD, Kosakovsky Pond SL (2010). Datamonkey 2010: a suite of phylogenetic analysis tools for evolutionary biology.. Bioinformatics.

[66] Crooks GE, Hon G, Chandonia JM, Brenner SE (2004). WebLogo: a sequence logo generator.. Genome Res.

