# Supplementary material for: Sequence Polymorphism, Segmental Recombination and Toggling Amino Acid Residues within the DBL3X Domain of the VAR2CSA Placental Malaria Antigen
Source: PLoS One. 2012 Feb 9;7(2):e31565. doi: 10.1371/journal.pone.0031565 (PMC3276574; doi:10.1371/journal.pone.0031565)
Supplement: Figure S1 — Amino acid alignment of 79 var2csa DBL3x domains. Individual subdomains are denoted by arrows above alignment. Toggling sites are marked by a “T” above the relevant amino acid site. Motifs identified in this study and in Dahlback at al. [33] are denoted by black boxes. Amino acid residues are colored according to conservation. (PDF) [file pone.0031565.s001.pdf]

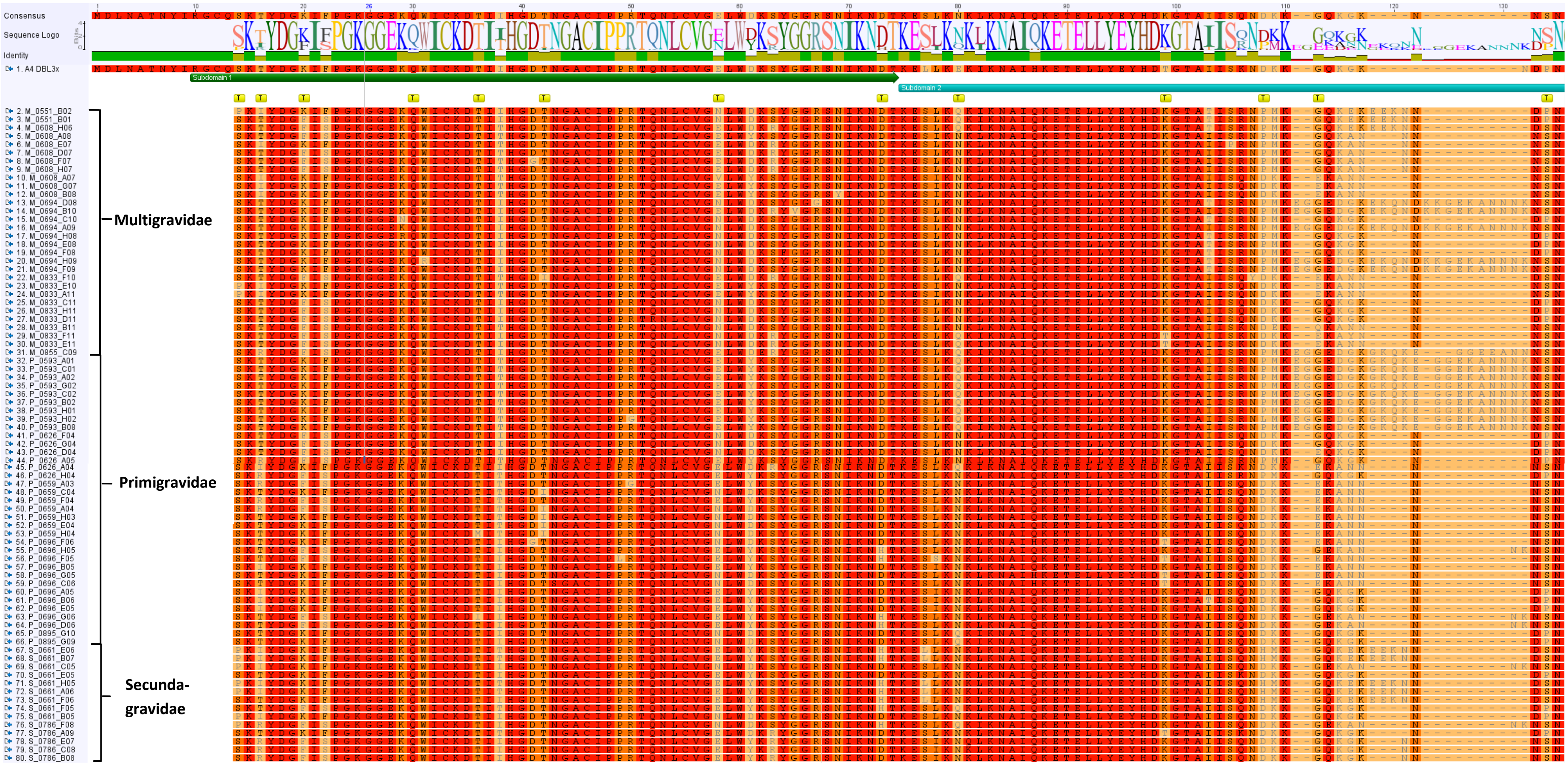

Supplementary Figure 1. Amino acid alignment of 79 var2csa DBL3x domains. Individual subdomains are denoted by arrows above alignment. Toggling sites are marked by a "T" above the relevant amino acid site. Motifs identified in this study and in Dahlback at al. [33] are denoted by black boxes. Amino acid residues are colored according to conservation.

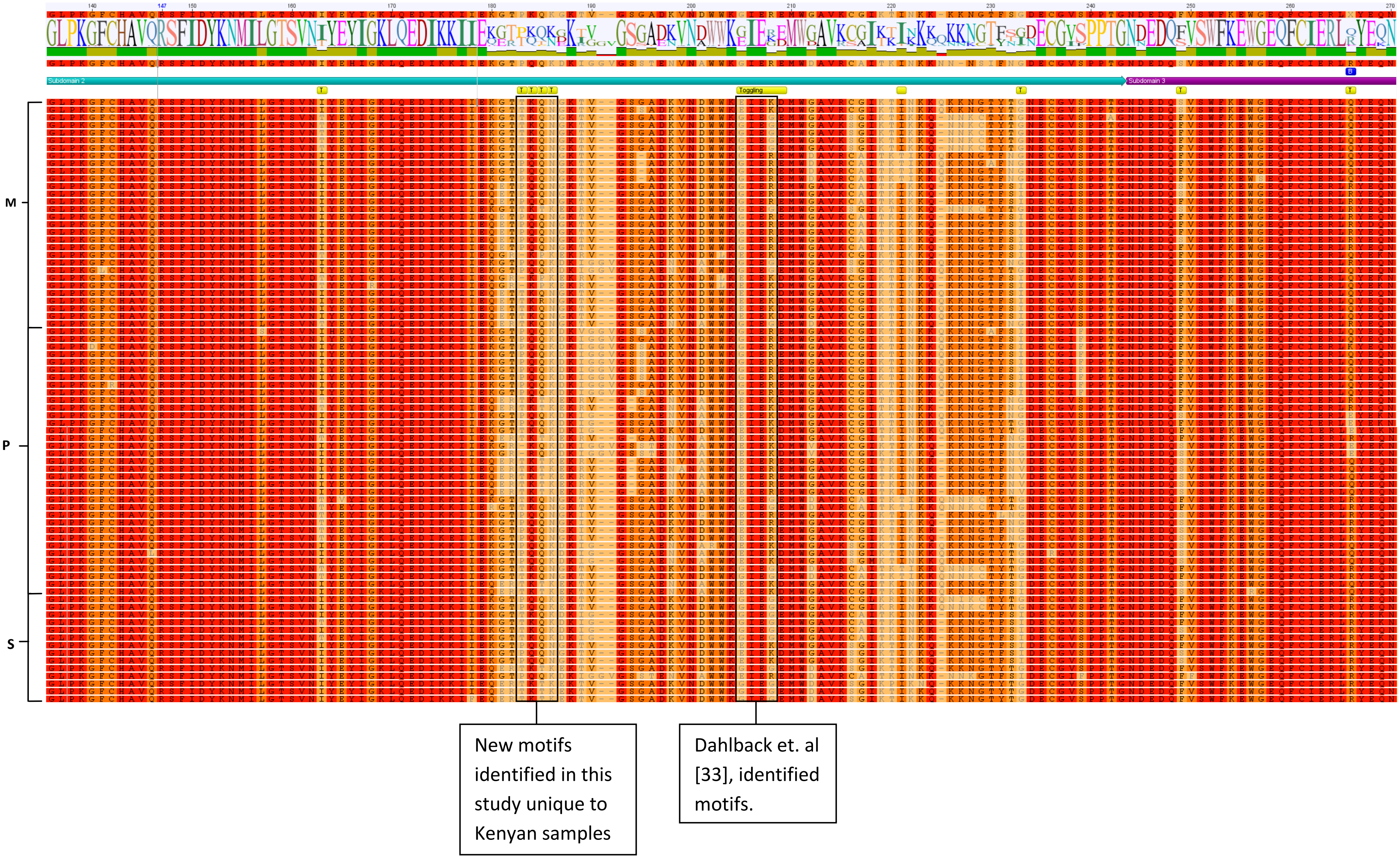
Subdomain 2
Subdomain 3
Toggling
M
P
S
New motifs identified in this study unique to Kenyan samples
Dahlback et. al [33], identified motifs.

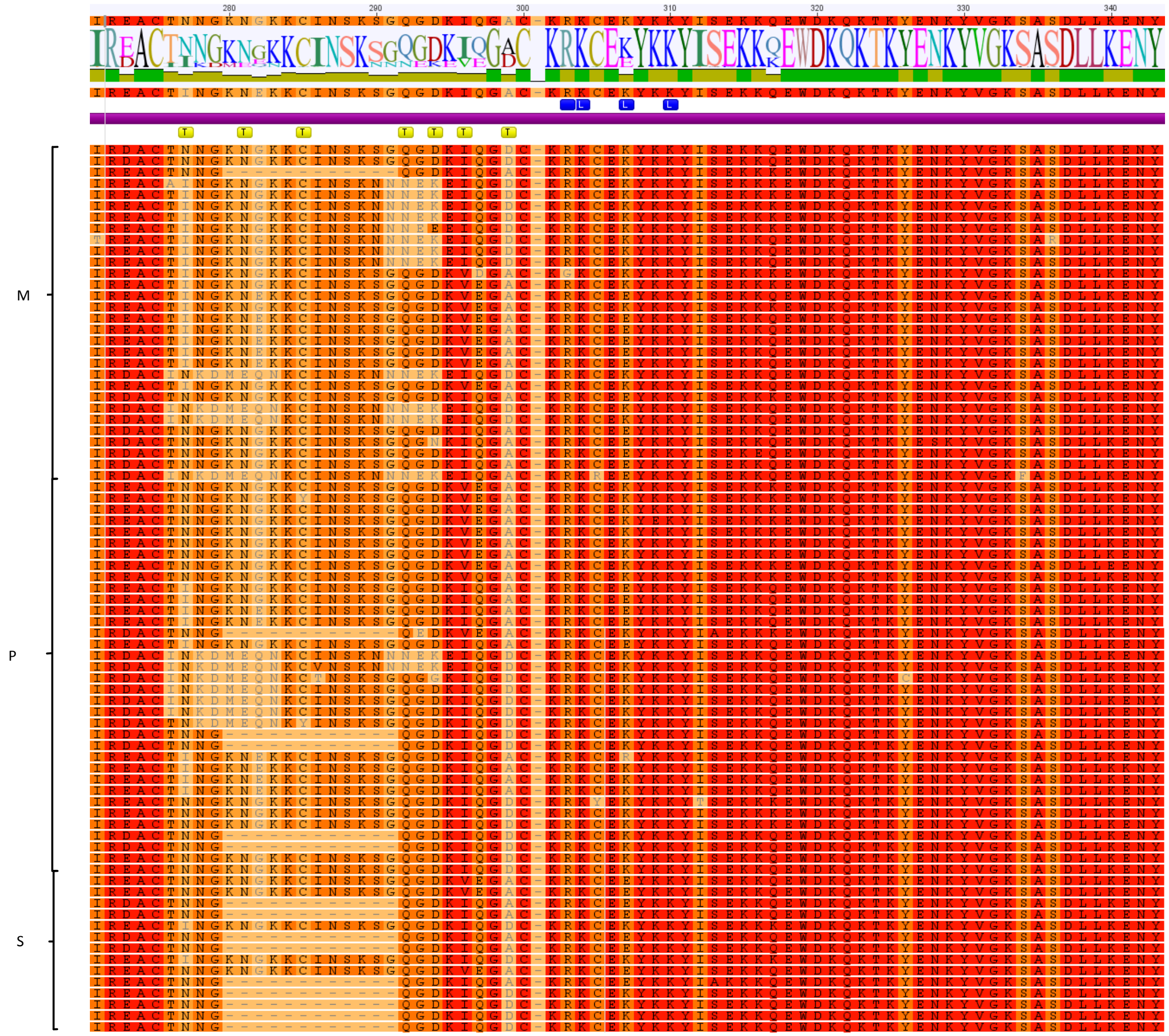
M
P
S
