# Supplementary material for: Sequence Polymorphism, Segmental Recombination and Toggling Amino Acid Residues within the DBL3X Domain of the VAR2CSA Placental Malaria Antigen
Source: PLoS One. 2012 Feb 9;7(2):e31565. doi: 10.1371/journal.pone.0031565 (PMC3276574; doi:10.1371/journal.pone.0031565)
Supplement: Table S1 — Relative proportions of toggling amino acids at each amino acid position. Amino acids found to toggle were stratified by gravidity. Relative proportions of occupying amino acids at each respective site within each group are represented as a percentage. Nine sites (shown in grey and denoted by a asterisk) were found to differ statistically significantly (c2, P<0.05) between the gravidity groups. (PDF) [file pone.0031565.s002.pdf]

Toggling Site	Primigravidae (N=35)				Multigravidae (N= 30)				aa Site
1	S	P			S	P			1232
	85.70%	14.30%			90.00%	10.00%			
2	R	I	**T***		R	I	**T***		1234
	18.40%	22.40%	**59.00%**		3.30%	23.30%	**73.30%**		
3	K	F			K	F			1238
	65.30%	34.70%			53.30%	46.70%			
4	Q	K			Q	K			1248
	93.80%	6.20%			86.70%	13.30%			
5	I	M	T		I	M	T		1254
	2.10%	2.10%	95.80%		0.00%	0.00%	100.00%		
6	T	**I***			T	**I***			1260
	89.60%	**10.40%**			96.70%	**3.30%**			
7	**N***	**E***	V		**N***	**E***	V		1276
	**31.40%**	**68.60%**	0.00%		**53.30%**	**43.30%**	3.30%		
8	**D***	**H***			**D***	**H***			1291
	**78.40%**	**21.60%**			**100.00%**	**2.00%**			
9	N	Q			N	Q			1298
	70.00%	30.00%			76.70%	23.30%			
10	K	T			K	T			1317
	86.30%	13.70%			93.30%	6.70%			
11	**D***	**P***	**H***		**D***	**P***	**H***		1326
	**41.40%**	**58.60%**	**0.00%**		**68.60%**	**19.60%**	**10.80%**		
12	Q	E	G		Q	E	G		1329
	0.00%	28.00%	72.00%		3.30%	26.70%	70.00%		
13	P	S			P	S			1336
	30.00%	70.00%			34.40%	68.60%			
14	I	T			I	T			1365
	73.50%	26.50%			70.00%	30.00%			
15	TKQN	PQQN	TKTK	PQQK	TKQN	PQQN	TKTK	PQQK	1385-1388
	16.00%	11.00%	29.00%	44%	38.00%	40.00%	7.00%	15.00%	
16	GIEGE	GIERE	EIEKD		GIEGE	GIERE	EIEKD		1407-1411
	28.00%	37.00%	35.00%		30.00%	40.00%	30.00%		
17	I	T			I	T			1424
	92.20%	7.80%			86.70%	13.30%			
18	G	I	V		G	I	V		1434
	50.00%	50.00%	0.00%		52.00%	38.00%	10.00%		
19	**S***	**F***			**S***	**F***			1450
	**44.00%**	**56.00%**			**20.00%**	**80.00%**			
20	**R***	**Q***	E		**R**	**Q**	E		1467
	**36.70%**	**63.30%**	0.00%		**52.90%**	**35.30%**	**0.00%**		
21	**N***	**I***			**N***	**I***			1478
	**74.50%**	**25.50%**			**40.00%**	**60.00%**			
22	N	E			N	E			1482
	80.60%	19.40%			86.20%	13.80%			
23	C	Y			C	Y			1486
	94.60%	5.40%			100.00%	0.00%			
24	N	Q			N	Q			1493
	41.40%	58.60%			40.00%	60.00%			
25	**D***	**K***	N		**D***	**K***	N		1496
	**56.70%**	**36.70%**	3.30%		**93.90%**	**4.10%**	2.00%		
26	I	V			I	V			1497
	76.00%	24.00%			65.60%	34.40%			
27	A	D			A	D			1500
	56.00%	44.00%			56.70%	43.30%			

Supplementary Table 1. Relative proportions of toggling amino acids at each amino acid position.
Amino acids found to toggle were stratified by gravidity. Relative proportions of occupying amino acids at each respective site within each group are represented as a percentage. Nine sites (shown in grey and denoted by a asterisk) were found to differ statistically significantly (χ^2 , $P < 0.05$) between the gravidity groups.
