# Supplementary material for: Sequence Polymorphism, Segmental Recombination and Toggling Amino Acid Residues within the DBL3X Domain of the VAR2CSA Placental Malaria Antigen
Source: PLoS One. 2012 Feb 9;7(2):e31565. doi: 10.1371/journal.pone.0031565 (PMC3276574; doi:10.1371/journal.pone.0031565)
Supplement: Table S2 — Summary table of recombination analysis via RDP4 [38] at a global level (all sequences) and individual samples (same patient clusters). Data shows recombination breakpoints related to the sequence of the transferred fragment (major parent) and sequence closely related to transferred fragment (minor parent) including resulting recombinant. For statistical analysis the Chi-square statistical test in RDP4 was used. (PDF) [file pone.0031565.s003.pdf]

Recombinant	Breakpoints		Major Parent	Minor Parent	Detection Method	p-value
	Begin	End				
**Global Analysis**						
M_0608_A07, B08, G07	4	377	M_0833_A11	M_0608_H07	RDP, MaxChi, Chimaera, SiScan, 3Seq	0.05
M_0694_A08, D07, E07	398	645	M_0608_H07	M_0608_H06	RDP, MaxChi, 3Seq	0.05
M_0833_H11,C11, F10	524	748	S_0661_E06	S_0661_B05	RDP, GENECONV, MaxChi, Chimaera, SiScan, 3Seq	0.05
M_0855_C09	327	861	P_0659_C04	P_0626_D04	GENECONV, MaxChi, Chimaera, 3Seq	0.05
P_0626_H04	410	942	P_0626_A05	P_0626_G04	MaxChi, Chimaera, 3Seq	0.05
S_0661_A06, H05	484	958	S_0661_E06	S_0661_C05	GENECONV, MaxChi, Chimaera, 3Seq	0.05
S_0661_F06	129	425	S_0661_C05	S_0661_E06	RDP, Chimaera, MaxChi, 3Seq	0.05
**Individual Analysis**						
M_0608_A07, B08	364	674	M_0608_H06	M_0608_E07	GENECONV, MaxChi, Chimaera, 3Seq	0.05
M_0608_A08	38	215	M_0608_G07	M_0608_F07	GENECONV, MaxChi, Chimaera, 3Seq	0.05
M_0608_D07	28	104	M_608_F07	M_0608_G07	GENECONV, MaxChi, Chimaera, 3Seq	0.05
M_0608_E07	105	935	M_0608_F07	M_0608_A07	GENECONV, MaxChi, Chimaera, 3Seq	0.05
M_0608_G07	272	614	M_0608_H06	M_0608_E07	GENECONV, MaxChi, Chimaera, 3Seq	0.05
M_0608_H06	284	674	M_0608_F07	M_0608_G07	GENECONV, MaxChi, Chimaera, 3Seq	0.05
M_608_H07	410	639	M_0608_B08	M_0608_D07	GENECONV, MaxChi, Chimaera, 3Seq	0.05
M_694_A09	370	687	M_0694_A09	M_0694_B10	BootScan, GENECONV, MaxChi, Chimaera, SiScan, 3Seq	0.05
M_694_C10, E08, F08, H08	281	717	M_0694_B10	M_0694_H09	MaxChi, Chimaera, SiScan, 3Seq	0.05
M_0833_A11, F10, H11	495	908	M_0833_F11	M_0833_C11	RDP, BootScan, GENECONV, MaxChi, Chimaera, SiScan, 3Seq	0.05
M_0833_C11	524	716	M_0833_A11	M_0833_F11	RDP, BootScan, GENECONV, MaxChi, Chimaera, 3Seq	0.05
M_0833_D11	121	392	M_0833_E10	M_0833_B11	RDP,BootScan, GENECONV, MaxChi, Chimaera, 3Seq	0.05
P_0593 No Recombination Detected						

P_0626_A05	277	367		P_0626_A04	GENECONV, MaxChi, SiScan, 3seq	0.05
P_0626_D04	409	808	P_0626_A05	P_0626_F04	BootScan, MaxChi, Chimaera, SiScan	0.05
P_0626_F04, G04, H04	256	535	P_0626_A04	P_0626_A03	BootScan, MaxChi, Chimaera, SiScan	0.05
P_0659_A04, H04	68	939	P_0659_A03	P_0659_E04	RDP, BootScan,GENECONV, MaxChi, Chimaera, SiScan, 3Seq	0.05
P_0659_C04	366	912	P_0659_H03	P_0659_A03	BootScan, MaxChi, Chimaera, SiScan, 3Seq	0.05
P_0696_B05	262	920	P_0696_B06	P_0696_C06	RDP, SiScan, MaxChi, 3Seq	0.05
P_0696_F05	265	870	P_0696_H05	P_0696_B05	BootScan,MaxChi, SiScan, 3Seq	0.05
P_0696_F06	506	842	P_0696_G05	P_0696_H05	GENECONV, MaxChi, BootScan,MaxChi, SiScan, 3Seq	0.05
S_0661_A06	218	484	S_0661_B05	S_0661_C05	GENECONV, MaxChi, BootScan, 3Seq	0.05
S_0661_B05	219	832	S_0661_C05	S_0661_F05		0.05
S_0661_B07, E06, F05, F06	435	913	S_0661_A06	S_0661_C05	RDP, GENECONV, BootScan, MaxChi, Chimaera, 3Seq	0.05
S_0661_C05	597	689	S_0661_E06	S_0661_B05	RDP, GENECONV,Chimaera, SiScan, 3Seq	0.05
S_0661_E05	219	483	S_0661_E05	S_0661_B05	GENECONV,BootScan,MaxChi,3Seq	0.05
S_0786_A09	358	786	S_0786_C09	S_0786_F08	GENECONV,MaxChi, Chimaera, 3Seq	
M_0551 only two seqs.						

Table S2: Summary table of recombination analysis via RDP4 [38] at a global level (all sequences) and individual samples (same patient clusters). Data shows recombination breakpoints related to the sequence of the transferred fragment (major parent) and sequence closely related to transferred fragment (minor parent) including resulting recombinant. For statistical analysis the Chi-square statistical test in RDP4 was used.
